# Comparison of Intraoperative Blood Loss in Monopolar Transurethral Resection of the Prostate With and Without Two Weeks of Preoperative Dutasteride

**DOI:** 10.7759/cureus.80493

**Published:** 2025-03-12

**Authors:** Aurangzeb Shaukat Ali, Moin Anwar, Mubashar Abrar, Muhammad Farhan Azeem, Muhammad Bilal, Waqar Ali, Muhammad Rehan Idrees, Fatima Ashraf

**Affiliations:** 1 Urology, Allied Hospital, Faisalabad Medical University, Faisalabad, PAK; 2 General Surgery, Allied Hospital, Faisalabad Medical University, Faisalabad, PAK; 3 Urology, Madinah Teaching Hospital, Faisalabad, PAK; 4 Urology and Transplantation, Allied Hospital, Faisalabad Medical University, Faisalabad, PAK; 5 General Practice, Sehat Clinic, Faisalabad, PAK

**Keywords:** benign prostatic hyperplasia, dutasteride, intraoperative blood loss, monopolar turp, preoperative hemostasis

## Abstract

Introduction: Monopolar transurethral resection of the prostate (TURP) is a common surgical procedure for benign prostatic hyperplasia, often associated with significant intraoperative blood loss. Dutasteride, a 5-alpha reductase inhibitor, has been recommended to reduce perioperative bleeding by decreasing vascularity within the prostate.

Objective: The purpose of this study was to investigate the impact of pre-operative administration of dutasteride for a duration of two weeks on the reduction of intra-operative blood loss in patients undergoing monopolar TURP.

Method: This prospective, two-armed, quasi-experimental study enrolled 132 patients based on the specified inclusion criteria. Patients who fulfilled the criteria for monopolar TURP were administered 0.5mg dutasteride for two weeks prior to their TURP procedure. Afterward, these patients were admitted to the hospital ward and underwent the necessary preparations for the surgery. During the surgical procedure, the intra-operative irrigation fluid was quantified and collected and the hemoglobin (Hb) was tested. The amount of blood loss was then determined using an appropriate equation. The assessment of blood loss was conducted using several indicators, including the analysis of irrigation fluid, the measurement of Hb levels in the irrigation fluid, the preoperative Hb levels, and the weight of the resected tissue.

Result: A significant decline in blood loss was observed in the interventional group in comparison to the control group. The average blood loss observed in Group A was 296ml, whereas in Group B it was 370ml. Furthermore, the blood loss per gram was found to be 11.7ml/g in Group A and 14.7ml/g in Group B. The mean operative time for Group A was recorded as 42 minutes, while Group B had a mean operative time of 49 minutes.

Conclusion: The study's findings indicate a significant superiority of administering dutasteride before surgery for a duration of two weeks. A substantial reduction in both intraoperative blood loss and blood loss per gram is seen in patients who underwent monopolar TURP with dutasteride.

## Introduction

The presence of excessive cellularity in both the epithelial and stromal components is a contributing factor to the development of benign prostatic hyperplasia (BPH) [1]. Several theories suggest that multiple components cause progression to BPH including stem cell replication, anti-apoptotic activity, embryonic stromal awakening and hormonal pathophysiology, the latter being the most widely accepted explanation [2]. Testosterone converts into dihydrotestosterone (DHT) under the influence of the 5-α-reductase enzyme mainly present in the prostate tissue. Androgen binds to androgen receptor. One of the isomers of androgen receptors is present in the prostatic tissue [3]. DHT binding to the receptor forms a protein complex that later causes translational modification and phosphorylation.

Stem cells play a crucial role in maintaining prostatic cellular integrity by facilitating controlled hyperplasia [4]. It is seen that the stromal-to-epithelial ratio is affected by this proliferation if prolonged, causing it to rise from 2:1 to 5:1 in BPH. This hyper-proliferation of stromal to epithelial elements of prostate tissue leads to BPH [5]. A defect in cellular apoptotic activity when joined with hyper-proliferation of prostate tissue leads to an increase in hyperplasia [6]. The reawakening of prostatic stroma selectively happens in the transition-peri-urethral zone (TPZ) because abortive distal ducts literally require renovation which is done by mesenchymal stem cells as a response to degradation. This mechanism in TPZ reawakens inductive stroma which actually triggers an increase in a number of prostatic cells that leads to BPH [7].

Studies revealed that first-degree relatives of patients who underwent some surgery for BPH have a much higher risk of developing BPH. Previous works exhibit that patients with no documented case of BPH in first-degree relatives have a 17% chance of developing BPH in their lifespan compared to the 66% lifetime risk in patients who have this disease documented in first-degree relatives [8]. The ongoing BPH puts pressure on the urethra and leads to the narrowing of its lumen which eventually causes lower urinary tract symptoms (LUTS). More than 50% of the male population above the age of 60 and 80% of males above the age of 90 suffer from some degree of LUTS, which affects their daily lives [9]. The quantification of LUTS is accomplished with the use of the International Prostate Symptom Score (IPSS). This scoring system comprises a total of eight questions, with seven questions dedicated to evaluating LUTS and one question specifically designed to assess the individual's quality of life (QOL).

Patients presenting with LUTS are typically prescribed medicinal therapy. The prostate gland consists of smooth muscle and significant α-1-receptors that are located in the prostate gland, prostatic capsule, prostatic urethra, and trigone of the bladder. A commonly used first-line treatment for LUTS is α-blockers. They block the stimulation and smooth muscle contraction and significantly reduce voiding LUTS [10, 11]. DHT is the biologically active metabolite of testosterone and is associated with the proliferation of stromal and epithelial components, ultimately resulting in the development of BPH. The class of 5-α-reductase inhibitors encompasses finasteride and dutasteride, both of which function by inhibiting the enzymatic conversion of testosterone to DHT, resulting in a reduction in prostate weight. The approximate half-life of dutasteride is estimated to be around 35 days. The primary isozyme of 5-α-reductase, known as isozyme-II, is predominantly expressed in the prostate epithelium while isozyme-I is primarily found in the skin and liver. Dutasteride demonstrates inhibitory effects on both isozyme-I and isozyme-II. It has been observed to reduce the expression of vascular endothelial growth factor (VEGF) and mean vascular density (MVD), thereby exerting an inhibitory effect on angiogenesis in prostatic tissue. In all of its aspects, it diminishes the vascularity of the prostate and minimizes the occurrence of hematuria [12-15]. Patients who fail the conservative therapy, are left with surgical options. Monopolar transurethral resection of the prostate (TURP) is regarded as the gold standard modality for BPH patients. It is widely available, economical, and comes with a short learning curve. It significantly reduces LUTS and improves urinary flow rate (Qmax). However, hemorrhage is considered the most common complication of TURP. Patients may require blood transfusions [14-16].

Hemorrhage is a common complication of TURP due to surgical trauma. Blood loss in TURP patients is often estimated using subjective indicators such as irrigation fluid color, hematuria duration, catheterization length, transfusion need, and hospital stay. A more precise method involves measuring hemoglobin in irrigation fluid, irrigation volume, and preoperative hemoglobin levels [17, 18]. Dutasteride, when administered for two weeks preoperatively, may help reduce intraoperative blood loss, with its maximum effect observed within one to two weeks of a 0.5 mg daily dose [19]. Studies from Korea and Southeast Asia have shown that preoperative dutasteride reduces TURP-related bleeding and hospital stay [20]. Traditional blood loss estimation methods, such as hemoglobin and hematocrit differences, are unreliable. A novel and more accurate approach involves assessing hemoglobin levels in blood and irrigation fluid, though further research is needed to refine this method.

In developing countries, patients usually belong to low socio-economic status. Most of them have moderate-severe anemia. Furthermore, physiological aging, diminished levels of physical activity, and reduced physiological reserves are the reasons why some of the patients require post-op blood transfusions. To deal with such, we require methods to reduce peri-op blood loss. If dutasteride proves to be efficient in reducing blood loss during TURP, its use might become routine practice.

Objective of the study

The purpose of this study was to investigate the impact of pre-operative administration of dutasteride for a duration of two weeks on the reduction of intra-operative blood loss in patients undergoing monopolar TURP.

## Materials and methods

After receiving approval from the institutional ethics committee of Faisalabad Medical University (Approval No. 48ERC/FMU/2022-23/297), 132 male patients aged 40 to 80 years with lower urinary tract symptoms (LUTS) were enrolled based on predefined inclusion and exclusion criteria from July 2023 to December 2023. This prospective, two-armed, quasi-experimental study was conducted in the urology and kidney transplantation department of Allied Hospital, Faisalabad Medical University. Patients who met the eligibility criteria were assigned to one of two groups using an even-odd serial number allocation method. This method ensured an unbiased distribution of patients into the intervention and control groups, facilitating comparative analysis while maintaining the quasi-experimental nature of the study.

Patients included in the study were males aged 40 to 80 years with LUTS who had failed conservative management or had refractory urinary retention, regardless of age-related variations in prostatic enlargement. Eligible patients had a prostate weight between 40 and 80 grams, as assessed by a digital rectal examination (DRE), which revealed a firm prostate with a smooth surface and non-adherent mobile mucosa. Prostate-specific antigen (PSA) levels were considered within age-specific ranges: 0-2.5 ng/ml for ages 40-49, 0-3.5 ng/ml for ages 50-59, 0-4.5 ng/ml for ages 60-69, and up to 6.5 ng/ml for those above 70 years.

Patients were excluded if they had any blood disorder or coagulopathy causing abnormalities in hemoglobin, platelets, white blood cells, bleeding time, clotting time, prothrombin time (PT), activated partial thromboplastin time (aPTT), or international normalized ratio (INR). Those with a history of cerebrovascular accident (CVA) or myocardial infarction (MI), inability to tolerate spinal anesthesia, or ongoing use of antiplatelet or anticoagulation therapy were also excluded. Additional exclusion criteria included renal impairment with deranged renal function tests (RFTs), the presence of other lower urinary tract diseases such as untreated urinary tract infection (UTI), neurogenic bladder, vesical (bladder) stones, urethral stricture, or recurrent hematuria. Patients with a history of previous TURP or those who developed complications during the procedure were also not considered for inclusion.

Patients were assessed in the urology OPD and those who fell under the treatment line of TURP were prescribed dutasteride for two weeks and were admitted to the urology ward on the 13th day of medicine. Patients were examined in the ward and other protocols were performed the next day. After admission to the ward, written informed consent was taken and all preoperative requirements were obtained. The evaluation of each subject included demographic information, a general history, a detailed urological history, a physical exam, and investigations. Urological history includes lower urinary tract symptoms, hematuria, catheterization history, urinary tract infection, and any prostatic or urethral surgery. The physical examination consisted of a general examination, examination of the renal region, external genitalia, and digital rectal examination, whereas the investigations included a complete blood count, renal function tests, and serum PSA. Ultrasound of the kidneys, ureters, and bladder with residual volume was performed after voiding to evaluate the size, volume, and texture of the prostate.

The patients were divided into two groups: Group A and Group B. Group A patients underwent TURP with pre-operative dutasteride use for two weeks, while Group B patients underwent TURP without it. Even and odd serial numbers were used for categorization. Even serial numbers were assigned to patients in Group A, the intervention group, while odd serial numbers were assigned to patients in Group B, the control group. On the specified date, surgical procedures were performed. For consistency, the same experienced urologist performed all TURP procedures in the study using standardized surgical protocols, with patients under spinal anesthesia in the lithotomy position. After obtaining the findings from a urethro-cystoscopy, TURP was performed using monopolar diathermy, a resectoscope, and 5% dextrose irrigation fluid. Following the completion of the resection, hemostasis was achieved. Three-way Foley catheterization was performed, irrigation was initiated, and the resection time in minutes (min) was recorded.

A sample of irrigation fluid was collected for the purpose of quantification, and 10 mL was extracted using a syringe. The concentration of Hb in the irrigation fluid was examined, and subsequently, the amount of blood loss was determined using the following equation:

$\text{Calculated blood loss (mL)} = \frac{\text{Hb in irrigation fluid (g/dL)} \times \text{irrigation fluid volume (mL)}}{(\text{pre-operative Hb (g/dL)} \times 10^{3})}$

The weight of the resected tissue was measured using a weighing machine in grams (g), and the amount of blood loss per gram of prostate tissue was calculated as follows:

$Blood\,loss\,per\,gram = \frac{\text{Blood loss (mL)}}{\text{Resected tissue weight (g)}}$

All the data was noted on the designed proforma, processed, and analyzed using IBM SPSS Statistics (version 25, IBM Corp., Armonk, NY) and Microsoft Excel (Microsoft Corp., Redmond, WA).

## Results

Our study reveals a statistically significant reduction in blood loss among patients who underwent TURP following a two-week preoperative regimen of dutasteride. The baseline characteristics of the participants were largely comparable between the two groups (Table 1). The mean age in Group A was 60.7 ± 11.4 years, slightly lower than the 64.5 ± 13.96 years observed in Group B. Prostate weight showed minimal variation, averaging 62.22 ± 10.4g in Group A and 61.9 ± 10.6g in Group B. Preoperative hemoglobin levels were also relatively similar, with Group A having a mean Hb of 12.84±1.11g/dL compared to 12.34 ± 1.5g/dL in Group B. However, notable differences emerged in procedural variables. The volume of irrigation fluid used during TURP was lower in Group A (13590.9 ± 1080.9ml) than in Group B (14446.9 ± 1228.2ml), suggesting reduced intraoperative bleeding. Furthermore, hemoglobin content in the irrigation fluid was lower in Group A (0.27 ± 0.05g/dL) than in Group B (0.30 ± 0.04g/dL), reinforcing the observed trend of decreased blood loss with dutasteride administration.

**Table 1:** Independent two-sample t-tests were used to compare demographics and procedural variables

Variable	Group-A (Mean ± SD)	Group-B (Mean ± SD)	P-Value	Test Statistic (t)	Odds Ratio (OR)	95% CI
Age (years)	60.7 ± 11.4	64.5 ± 13.96	0.08	1.76	1.12	0.94-1.34
Prostate weight (grams)	62.22 ± 10.4	61.9 ± 10.6	0.67	0.42	0.96	0.85-1.09
Operative time (min)	41.16 ± 6.67	48.22 ± 6.73	<0.001	4.76	1.58	1.32-1.88
Pre-op Hb (g/dL)	12.84 ± 1.11	12.34 ± 1.5	0.09	1.84	0.87	0.74-1.03
Vol. of irrigation fluid (ml)	13590.9 ± 1080.9	14446.9 ± 1228.2	0.03	2.13	1.22	1.01-1.48
Hb in irrigation fluid (g/dL)	0.27 ± 0.05	0.30 ± 0.04	0.001	3.45	1.41	1.18-1.69
Resected tissue weight (g)	25.22 ± 5.9	26.22 ± 4.89	0.34	0.96	1.08	0.92-1.27

The amount of blood loss observed in patients belonging to Group A was measured to be 288 ± 74.68ml, whereas in Group B, it was found to be 359 ± 69.1ml. The duration of the surgical procedure was also decreased, with Group A patients undergoing TURP for an average of 41.16 ± 6.67 minutes, compared to 48.22 ± 6.73 minutes for Group B patients. The amount of blood loss per gram of resected tissue was found to be 11.38±1.36ml/g in Group A and 13.96±2.75ml/g in Group B. The findings of the study demonstrated that the administration of dutasteride resulted in a decrease in both intra-operative blood loss and blood loss per gram during TURP (Table 2).

**Table 2:** Independent two-sample t-tests were used to compare blood loss analysis in the two groups

Variable	Group A (Mean ± SD)	Group B (Mean ± SD)	P-value	Test statistic (t)	OR	95% CI
Blood loss (mL)	288 ± 74.68	359 ± 69.1	<0.001	5.21	1.75	1.46-2.09
Blood loss per gram (mL/g)	11.38 ± 1.36	13.96 ± 2.75	<0.001	4.67	1.89	1.53-2.31

One of the most compelling findings of this study was the significant reduction in operative time among patients who received preoperative dutasteride. On average, TURP was completed in approximately 41 minutes in Group A, compared to nearly 48 minutes in Group B. This shorter surgical duration likely contributed to the overall reduction in blood loss. Additionally, the volume of irrigation fluid required during the procedure was noticeably lower in Group A, further supporting the hemostatic benefits of dutasteride in minimizing intraoperative bleeding. Beyond its intraoperative advantages, dutasteride also appeared to enhance postoperative recovery. Patients in Group A experienced significantly faster catheter removal, with a mean duration of 2.1 ± 0.9 days, compared to 2.8 ± 1.1 days in Group B. Hospital stays followed a similar trend, with Group A patients being discharged earlier (3.2 ± 1.2 days) than those in Group B (4.0 ± 1.5 days). Moreover, postoperative complications were substantially lower in Group A, affecting only 18.2% of patients (n=12), whereas 36.4% (n=24) of those in Group B experienced adverse events. The benefits extended to the need for re-catheterization, which was significantly reduced in Group A (7.6%; n=5) compared to Group B (22.7%; n=15). These findings underscore the comprehensive advantages of preoperative dutasteride administration, not only in reducing intraoperative blood loss but also in promoting faster recovery, shorter hospital stays, and lower rates of complications and re-catheterization.

**Table 3:** Postoperative outcomes Z: Mann-Whitney U test, χ²: Chi-square test.

Variables	Group A	Group B	Test statistic	P-value
Catheter removal time (days, mean ± SD)	2.1 ± 0.9	2.8 ± 1.1	Z = -3.21	0.001
Hospital stay (days, mean ± SD)	3.2 ± 1.2	4.0 ± 1.5	Z = -2.89	0.004
Post-op complications (%)	12 (18.2%)	24 (36.4%)	χ² = 4.92	0.027
Recatheterization (%)	5 (7.6%)	15 (22.7%)	χ² = 5.44	0.019

## Discussion

BPH is one of the most common urological disorders that affects men who have surpassed the fifth decade of life. Several theories have been proposed to explain the causes of BPH, including stem cell replication, anti-apoptotic activity, embryonic stromal awakening, and hormonal pathophysiology. Among these theories, hormonal pathophysiology is considered to be the most appropriate explanation. Most of the patients are initially managed with conservative medicinal therapy. Pharmacotherapies are mostly consisting either α-adrenergic blockers or 5-α-reductase inhibitors or sometimes combination therapy. Several phytotherapies have been in use in Europe and the United States and are considered to as alternative therapies to pharmacologic agents. Several studies have evaluated the efficacy of* Serenoa repens* and *Cucurbeta pepo* in the treatment of BPH. A recent study by Crocerossa et al. on the gamma-cyclodextrin-curcumin complex showed that it significantly reduces LUTS and improves the quality of life [21].

Patients who do not exhibit a positive response to therapy are eventually offered the option of undergoing surgical procedures. A systematic review and meta-analysis with 15 studies involving 6659 patients exhibited the comparison and effectiveness of different surgical options for BPH. According to the literature, robot-assisted simple prostatectomy (RASP) is preferred over open simple prostatectomy (OSP) and laparoscopic simple prostatectomy (LSP) as the former has a very low error or failure rate. However, limited availability and high costs make it inaccessible to many patients and payers. OSP presents with significant post-op complications while LSP with a long learning curve requires solid laparoscopic skills. TURP, an electrosurgical procedure, continues to be considered the preferred and most common surgical treatment option due to its widespread availability and cost-effectiveness. According to a study conducted by Worthington in 2017 [22], TURP has been the standard surgery for BPH for 40 years, with approximately 25,000 procedures performed annually. Although generally successful, this procedure has well-documented risks, the most common being intraoperative bleeding [22].

In the prostate, the enzyme 5-α-reductase facilitates the conversion of testosterone into DHT. It exhibits an affinity for its specific receptors located within prostatic tissue, thereby initiating a chain of events that ultimately leads to the development of BPH. DHT additionally stimulates a rise in VEGF gene expression. The increased level of VEGF expression leads to an elevation in MVD within the sub-urethral prostatic tissue, thereby enhancing the chance of experiencing hematuria. This clarifies the rationale behind the occurrence of hematuria in untreated patients with BPH. Dutasteride is classified as a medication that exerts a permanent inhibitory effect on the enzyme 5-α-reductase, resulting in a substantial decrease in DHT levels. Consequently, this reduction in DHT levels leads to a decrease in VEGF and MVD [23, 24].

In our experimental study, dutasteride was administered to the participants undergoing TURP two weeks prior to the surgical procedure. This decision was based on the assumption that this specific time frame would adequately diminish MVD and angiogenesis within the prostatic tissue. Subsequently, our study's findings provided support for this hypothesis.

A systematic review and meta-analysis by Kloping et al. demonstrates a statistically significant decrease in operative time [17]. A trial conducted by Rahman et al. exhibited results in favor of dutasteride regarding operation time [20]. In our study, findings indicated that the utilization of dutasteride two weeks prior to TURP surgery results in a reduction in both blood loss and blood loss per gram.

Limitations of the study

Our study has several limitations, including its single-center design with a restricted sample size, which may limit the external validity and generalizability of the findings. A limitation is the relatively short duration of dutasteride administration (two weeks) prior to TURP, which may not fully elucidate its long-term hemostatic efficacy; while our study specifically evaluated its short-term impact on intraoperative blood loss, an extended preoperative administration period (e.g., three months) could potentially enhance its hemostatic benefits. The study did not incorporate an assessment of long-term postoperative complications, including retrograde ejaculation, which may necessitate further prospective longitudinal evaluation. The inclusion of younger patients with refractory urinary retention, although meeting predefined eligibility criteria, may not be entirely representative of the conventional BPH cohort. To mitigate potential biases, we implemented stringent inclusion and exclusion criteria, maintained standardized surgical techniques, and ensured uniform preoperative evaluations across all participants. Future large-scale, multicenter, randomized controlled trials with extended dutasteride administration durations and long-term follow-up are warranted to substantiate and refine our findings.

## Conclusions

This prospective, two-armed, quasi-experimental study demonstrates that a two-week preoperative course of dutasteride significantly reduces intraoperative blood loss in patients undergoing TURP. Beyond its hemostatic benefits, dutasteride also contributes to a decrease in the volume of irrigation fluid used and shortens the duration of surgery, both of which are crucial for optimizing procedural efficiency and patient outcomes. Furthermore, postoperative recovery is notably improved, as evidenced by shorter catheterization times, reduced hospital stays, and a lower incidence of complications and re-catheterization.

These findings highlight the potential of dutasteride as a valuable preoperative intervention for TURP, offering both surgical and recovery advantages. While the observed differences in perioperative parameters are statistically significant, further large-scale studies and randomized controlled trials are needed to validate these findings and establish Dutasteride as a standard pre-TURP treatment in clinical practice.
